# Persistent Odynophagia 27 Days After Emergent Intubation

**DOI:** 10.5811/cpcem.25018

**Published:** 2024-12-17

**Authors:** Richard White, Kaitlyn Mander, Christian A. Koziatek, Sanjay Mohan

**Affiliations:** * NYU Grossman School of Medicine, Ronald O. Perelman Department of Emergency Medicine, New York, New York; † Bellevue Hospital Center, Department of Emergency Medicine, New York, New York; ‡ Geisinger Community Medical Center, Emergency Services PC, Scranton, Pennsylvania; § New York University Grossman Long Island School of Medicine, Department of Emergency Medicine, Mineola, New York

**Keywords:** intubation, foreign body, dentures, odynophagia

## Abstract

**Case Presentation:**

We describe a case of persistent odynophagia due to a retained foreign body 27 days after emergent intubation.

**Discussion:**

Dentures constitute a potential esophageal foreign body and warrant special consideration during airway management. Odynophagia, dysphagia, and changes in phonation should prompt consideration of retained esophageal foreign bodies, especially in the post-intubation setting.

## CASE PRESENTATION

**Image 1:**
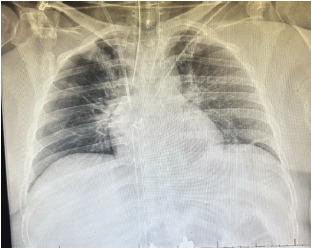
Post-intubation chest radiograph.

A 41-year-old female, with no past medical history, presented to the emergency department (ED) with a chief complaint of odynophagia. The patient initially presented to the ED 29 days prior for back pain after a motor vehicle accident and was found to have an unstable L1 burst fracture. She underwent an uncomplicated L1 surgical fixation after routine endotracheal intubation on the day of presentation and was extubated without any untoward effects. Postoperatively, on hospital day two, computed tomography (CT) of the chest revealed bilateral pulmonary emboli. No foreign bodies were noted in the esophagus or trachea. Later the same day, the patient suffered a cardiac arrest and was emergently intubated. Post-intubation single-view portable chest radiographs did not reveal any obvious foreign bodies in the thoracic cavity (Image 1).

She was eventually weaned off mechanical ventilation on hospital day six with a normal mental status and reports of minor odynophagia immediately post-extubation. She was ultimately discharged to a rehabilitation facility on hospital day 23, on a regular diet, without any subsequent imaging performed.

She returned to the ED six days later with progressively worsening odynophagia since discharge. She noted discomfort with swallowing liquids and solids but denied emesis, changes in phonation, or difficulty breathing. Vital signs were unremarkable. Physical examination revealed an age-appropriate female in no apparent distress and otherwise tolerating her secretions. Her uvula was midline, and an oropharyngeal examination revealed no evidence of infection or trauma. Her neck was supple, and there was no pain upon palpation of the neck. No upper airway or abnormal breath sounds were noted.

**Image 2:**
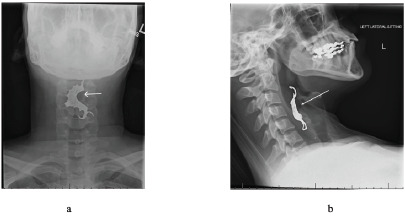
Anterior-posterior (a) and lateral (b) radiographic views of a foreign body (white arrows) in the hypopharynx.

Two-view cervical roentgenograms ultimately revealed a foreign body in the hypopharynx (Image 2). Otolaryngology was consulted. Using flexible nasopharyngoscopy, they visualized a dental plate over the larynx at the level of the cervical esophagus. The patient was emergently taken to the operating room to remove the foreign body and was cleared for discharge to a rehabilitation facility three days later without any changes in phonation, difficulty breathing or swallowing, and she was able to tolerate food and liquids by mouth.

## DISCUSSION

After animal bones, dentures are the second most commonly ingested foreign body and account for approximately 4–18% of esophageal foreign bodies.^1^ Dental prosthetic dislodgement, in general, displays high rates of endoscopic or surgical intervention due to its tendency to become impacted or lead to perforation.^2^ It is worth noting that dentures, whether total or partial, may or may not be visualized with standard roentgenograms as some are radiolucent.^3^

Although emergent intubation was suspected to be the sentinel event leading to the patient’s presentation, other etiologies including chest compressions during cardiopulmonary resuscitation leading to dental plate dislodgement, extubation, or unintentional swallowing of the foreign body at any point during the prolonged hospitalization were considered. Dislodgement and retention of dental devices in the context of airway management has been reported previously, but it is worth noting again as a visual reminder of this avoidable and potentially catastrophic mishap during intubation, as well as the duration of time this patient went without identifying the retained foreign body.^3^–^5^

Clinicians should be attuned to the risks of dental hardware that may be dislodged unintentionally, particularly in emergent situations such as rapid sequence intubation or cardiac arrest. It is critical to maintain a high index of suspicion for foreign body ingestion in specific patient populations, such as those with psychiatric disorders or cognitive delay, as these groups carry higher rates for such events.^5^ Furthermore, advanced imaging (such as computed tomography) should be considered to definitively evaluate for the presence of dental hardware, as roentgenograms may miss radiolucent foreign bodies.

**CPC-EM Capsule**

What do we already know about this clinical entity?

*Rapid sequence intubation poses the risk of foreign body aspiration*.

What is the major impact of the image(s)?

*Dislodged dental foreign bodies should be in the differential for patients presenting with odynophagia after recent airway management*.

How might this improve emergency medicine practice?

*Prior to intubation, assessing for and documenting the presence of dental prosthetics is essential to minimize the risk of foreign body aspiration*.

This case is particularly relevant for emergency, critical care, and anesthesia clinicians whose scope of practice includes airway management, especially in emergent scenarios. Specifically, it highlights the importance of assessing for and documenting the presence of dental prosthetics before and after any procedure that risks dislodgement. Dislodged dental foreign bodies should be in the differential for patients presenting with odynophagia after recent airway management.
